# A Ménage à trois: NLRC5, immunity, and metabolism

**DOI:** 10.3389/fimmu.2024.1426620

**Published:** 2024-07-05

**Authors:** Fabienne Brunschwiler, Surender Nakka, Jessica Guerra, Greta Guarda

**Affiliations:** Università della Svizzera Italiana (USI), Faculty of Biomedical Sciences, Institute for Research in Biomedicine, Bellinzona, Switzerland

**Keywords:** NLRC5, CIITA, MHC, butyrophilins, Vγ9Vδ2 T cells, phosphoantigens

## Abstract

The nucleotide-binding and oligomerization domain-like receptors (NLRs) NLR family CARD domain-containing protein 5 (NLRC5) and Class II Major Histocompatibility Complex Transactivator (CIITA) are transcriptional regulators of major histocompatibility complex (MHC) class I and class II genes, respectively. MHC molecules are central players in our immune system, allowing the detection of hazardous ‘non-self’ antigens and, thus, the recognition and elimination of infected or transformed cells from the organism. Recently, CIITA and NLRC5 have emerged as regulators of selected genes of the butyrophilin (*BTN*) family that interestingly are located in the extended MHC locus. BTNs are transmembrane proteins exhibiting structural similarities to B7 family co-modulatory molecules. The family member BTN2A2, which indeed contributes to the control of T cell activation, was found to be transcriptionally regulated by CIITA. NLRC5 emerged instead as an important regulator of the *BTN3A1*, *BTN3A2*, and *BTN3A3* genes. Together with BTN2A1, BTN3As regulate non-conventional Vγ9Vδ2 T cell responses triggered by selected metabolites of microbial origin or accumulating in hematologic cancer cells. Even if endogenous metabolites conform to the canonical definition of ‘self’, metabolically abnormal cells can represent a danger for the organism and should be recognized and controlled by immune system cells. Collectively, new data on the role of NLRC5 in the expression of BTN3As link the mechanisms regulating canonical ‘non-self’ presentation and those marking cells with abnormal metabolic configurations for immune recognition, an evolutionary parallel that we discuss in this perspective review.

## Introduction

Nucleotide-binding and oligomerization domain (NOD)-like receptors (NLRs) are best known for their role in the detection of specific danger-associated molecular patterns (DAMPs) and pathogen-associated molecular patterns (PAMPs) that has been thoroughly discussed elsewhere (1, 2). In this perspective review, we instead focus on the transcriptional regulatory functions of NLR family CARD domain-containing protein 5 (NLRC5) and Class II Major Histocompatibility Complex Transactivator (CIITA). Through a molecular platform called ‘MHC-enhanceosome’, NLRC5 and CIITA regulate the constitutive and the interferon-induced transcription of major histocompatibility complex class I (MHCI) and II (MHCII) genes, respectively (3, 4). MHC molecules are the core of vertebrates’ immune system, enabling the distinction between ‘self’ and ‘non-self’ and as such are central to the function of different immune subsets, promoting immune activation or tolerance.

Intriguingly, recent findings reveal unappreciated parallels between the presentation to immune cells of ‘non-self’ molecules and metabolites derived from deregulated cell metabolism. Metabolism, the ensemble of processes that fulfill cellular bioenergetic and biosynthetic needs (5), is central to cellular function and can be adapted to respond to the cellular needs. In some cases, however, it can also represent a threat to the organism as alterations in metabolic configurations underlie – for example – malignant transformation (6, 7). It is conceivable that immunity evolved to identify metabolically abnormal cells, despite conforming to the classic definition of ‘self’. In this review, we will discuss transcriptional NLRs, with particular attention on the emerging ability of NLRC5 to regulate the transcription of *butyrophilins* (*BTNs*) that are key to reveal an altered mevalonate metabolism to immune cells (7–9).

## NLRC5 and CIITA; expression and domain organization

CIITA, the master regulator of MHCII genes, can be found in different forms according to the cell type-specific promoters used for its transcription. The form I features an N-terminal CARD effector domain followed by all characteristic NLR domains; the nucleotide binding domain (NBD), the helix domain 1 (HD1), the winged helix domain (WHD), the helix domain 2 (HD2), and a short stretch of leucine-rich repeats (LRRs). Instead, forms III and IV, the latter induced by interferon, lack the CARD (3, 10), while maintaining all the other domains.

NLRC5, the transactivator of MHCI genes, shows the typical domain organization of the other NLRs and is the largest member of the NLR family (4, 11). At the N-terminus it exhibits an ‘untypical’ CARD (uCARD), while the C-terminus is characterized by a long stretch of LRRs, which is the longest among NLRs. NLRC5 is largely regulated by interferons, both type I and II; accordingly, two signal transducer and activator of transcription (STAT) binding sites have been identified in its promoter (12, 13). Furthermore, expression of NLRC5 via peroxisome proliferator-activated receptor γ isoform 1 (PPARγ1), the master regulator of adipogenesis and lipid metabolism, has been reported (14).

While CIITA is almost exclusively found in antigen-presenting cells (APCs) and appears to be equally distributed between the cytosol and the nucleus, NLRC5 is broadly expressed and resides mostly in the cytoplasm with a small fraction shuttling to the nucleus, (13, 15–19). In the nucleus, both NLRC5 and CIITA are recruited to the promoter of MHC and related genes through the MHC-enhanceosome (11, 18, 20, 21). Interestingly, both NLRC5 and CIITA do not show DNA binding motifs in their sequence, corroborating the importance of the interaction with MHC-enhanceosome components for their function (18, 22). Once occupying the promoter, these NLRs promote the transcription of target genes.

## Function of NLRC5 and CIITA in antigen presentation

**Figure 1:**
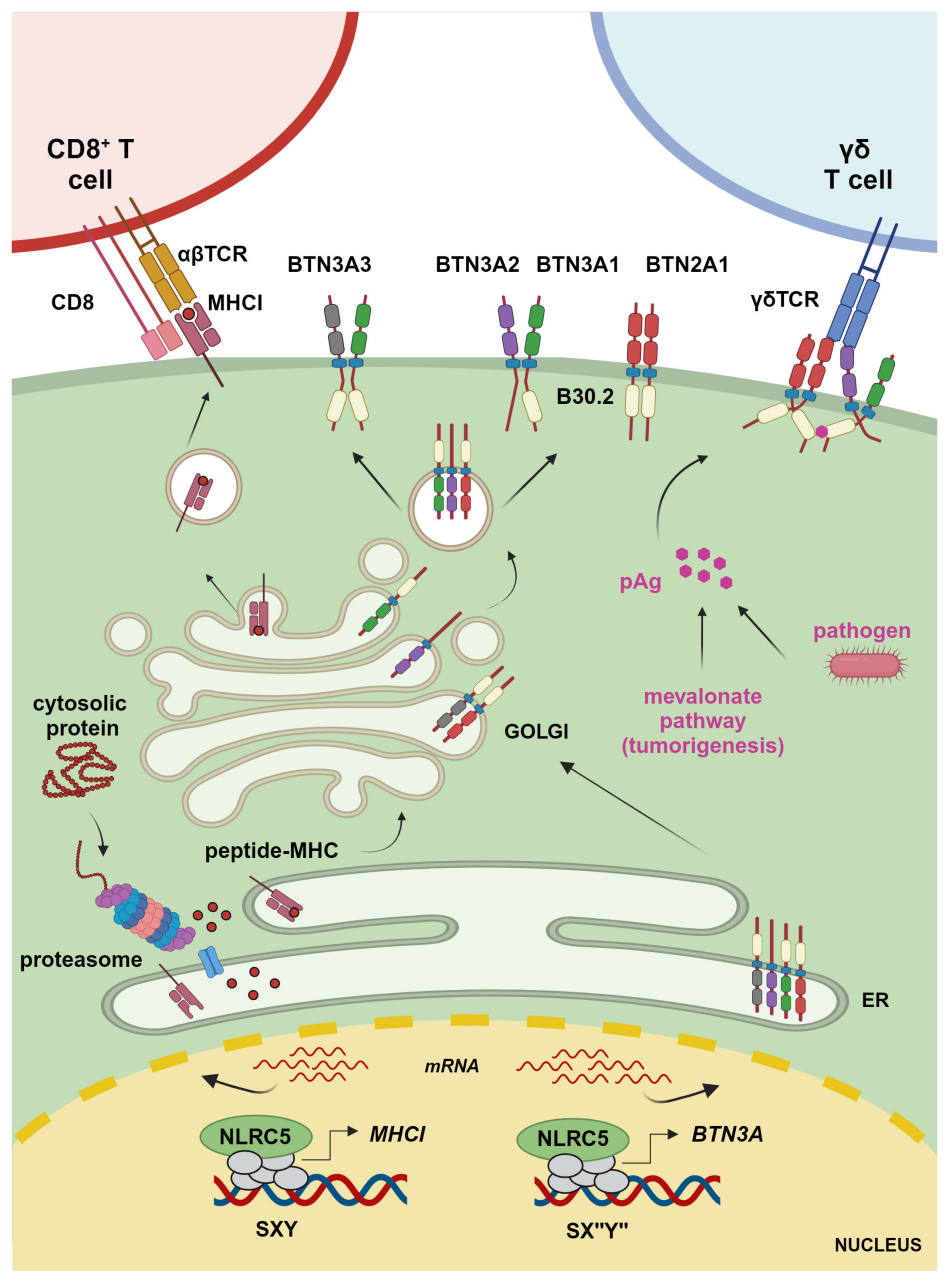
Transcriptional regulation of MHCI and BTN3A1–3 molecules. The transcriptional regulator NLRC5 translocates to the nucleus, where it binds to the SXY module of *MHCI* and *BTN3A* gene promoters along with a complex known as MHC-enhanceosome. NLRC5 recruits then chromatin modifiers, transcriptional elongation, and initiation factors to promote transcription of these target genes. MHCI molecules present proteasome-derived endogenous peptides to CD8^+^ T cells, whereas BTN3A1 binds – through the intracellular B30.2 domain – to pAgs accumulating due to infection or a hyperactive mevalonate pathway. This results in a conformational change inducing the interaction between the intracellular domain of BTN3A1, present as BTN3A1 homomer or heteromer with BTN3A molecules, and BTN2A1 and enabling the interaction between BTN2A1 and the TCR to mediate γδ T cell activation (figure was created by www.biorender.com).

T lymphocytes constitute an essential arm of adaptive immunity. CD4^+^ T cells, which exert modulatory functions, express a T cell receptor (TCR) recognizing the cognate peptide in the context of MHCII molecules. MHCII glycoproteins are expressed on APCs and display peptides derived from phagocytosis or autophagy (20, 23). Instead, CD8^+^ T lymphocytes kill target cells upon detection of the cognate antigen presented onto classical MHCI molecules (**Figure 1**). Classical MHCI proteins are ubiquitously expressed and present proteasome-derived peptides, including pathogen-derived or tumor antigens in infected or transformed cells, respectively (20, 23). In fact, T cells are essentially selected to react toward ‘non-self’, while recognition of ‘self’-peptides is limited to the regulatory subsets (24). Further, ‘unconventional’ T cells are restricted to non-classical MHCI molecules, which bind to peptides or specific biochemical structures of microbial or host-derived origin, eliciting effector or regulatory responses by unconventional T cells (25–27). Therefore, MHC molecules are central to our immune system, allowing to discriminate between ‘self’ and ‘non-self’.

CIITA regulates the expression of MHCII, which is composed of an alpha chain and a beta chain. In humans, three distinct MHCII molecules known as HLA-DR, HLA-DP, and HLA-DQ are encoded within the ‘MHC locus’, found on the short arm of chromosome 6. In mice instead, the ‘MHC locus’ located on chromosome 17 comprises two MHCII molecules, H2-A and H2-E. Moreover, CIITA regulates the expression of genes involved in MHCII loading, such as *HLA-DM*, *HLA-DO*, and *CD74* (28–30). CIITA is therefore a key player in coordinating immunity, as shown by the effects of inactivating CIITA mutations that lead to an immunosuppressed condition known as *bare lymphocyte syndrome* (BLS) (31).

The expression of classical MHCI glycoproteins, which are composed of an alpha chain and the invariant β-2-microglobulin (β2m), is largely regulated by NLRC5 (13, 18, 32, 33). Classical MHCI genes in humans are *HLA-A*, *HLA-B*, and *HLA-C*, whereas the murine genes are *H2-K*, *H2-D*, and *H2-L* and are located within the ‘MHC locus’. NLRC5 also regulates the human non-classical MHCI genes *HLA-E*, *HLA-F*, and *HLA-G* and murine *H2-M*, *H2-T*, and *H2-Q* family members as well as the expression of genes involved in the MHCI pathway, such as *β2m*, selected proteasome subunits, and *transporter associated with antigen processing 1 (TAP1)*, an essential molecule for antigen loading onto MHCI (34). Given its important function in the MHCI pathway, NLRC5 has been increasingly appreciated for its major role in regulating cytotoxic T cells toward cancerous cells (18, 33, 35–42).

Of note, while overexpression of NLRC5 does not induce MHCII expression, CIITA overexpression has been shown to induce MHCI transcription (43–46). However, *in vivo*, endogenous NLRC5 and CIITA show non-redundant functions (33, 34). This supports the idea that, due to the similar elements of the MHCI and MHCII promoters, overexpression of CIITA can lead to misleading results. In the following section, we will discuss in detail similarities and differences of MHC genes’ promoters and introduce in parallel the composition of the MHC-enhanceosome.

## MHC promoter

**Figure 2:**
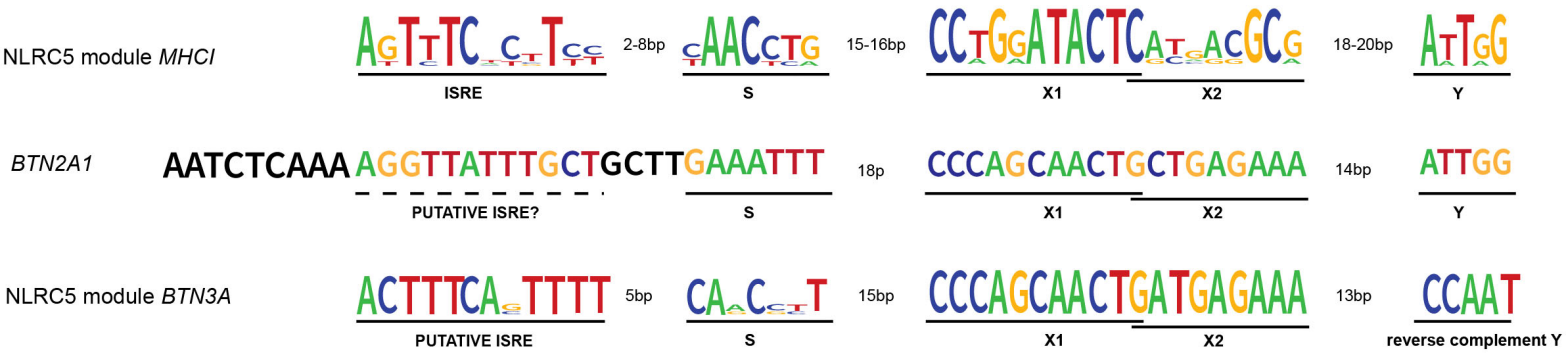
NLRC5’s transcription modules and *BTN2A1* promoter. NLRC5 module from MHCI genes’ promoters: MHCI genes’ promoter presents the typical SXY module crucial for the binding of the enhanceosome and the recruitment of NLRC5. The X box is then further divided into X1 and X2. Upstream of the SXY motif, there is the interferon-stimulated regulatory element (ISRE), which mediates the binding of IRF1. NLRC5 module from *BTN3A* genes’ promoters: as for the MHCI genes, the promoter of *BTN3A* genes shows the presence of an SXY module with the peculiarity of a reverse complement Y motif. Interestingly, the alignment performed using SnapGene software (www.snapgene.com), shows the presence of a putative ISRE at the distance and orientation expected for MHCI genes’ promoter. *BTN2A1* gene shows an SXY module with a Y motif in the same orientation as found in MHCI genes. However, our alignment did not show the presence of a highly conserved ISRE motif upstream of the SXY module. The logo for the consensus sequence of MHCI genes was created using http://weblogo.berkeley.edu (47) giving as input the respective ISRE, S, X, and Y sequences of *HLA-A*, *B*, *C*, *E*, *F*, and *G* genes. The same tool was used to generate the consensus for *BTN3A* genes giving as input the ISRE, S, X, and Y sequences of *BTN3A1–3* genes. All the genomic sequences were retrieved from the genome browser (genome.ucsc.edu).

The promoter region of both MHCI and MHCII genes contains the SXY module, which consists of the S box, the X box further divided into X1 and X2, and the Y box, altogether responsible for the binding of the enhanceosome and the recruitment of the matching NLR (**Figure 2**) (21, 34, 48–50). Interestingly, both sequence and stereo-specific position of the boxes forming the SXY motif are conserved and crucial for the constitutive and induced expression of MHCI and MHCII genes (34, 51, 52). The X1 and Y boxes are bound by regulatory factor X (RFX) and nuclear factor Y (NF-Y) complexes, respectively (49, 53–55). The X2 box is bound by CREB/ATF1(cAMP response element-binding protein) family members (44, 56, 57). Interestingly, the S-box is important for both CIITA- and NLRC5-mediated transactivation and its sequence dictates the specificity of NLRC5 for MHCI (11, 34, 49). Despite this knowledge, the S-box binding factor(s) is still unknown.

Upstream of the SXY box, the promoter of MHCI genes contains two additional regulatory sequences; the enhancer A and the interferon-stimulated responsive element (ISRE) bound by NF-κB and IRF1, respectively (**Figure 2**) (44, 54, 58, 59). The latter contributes to the interferon (IFN) type I- and type II-induced expression of MHCI genes.

The expression of MHCI and MHCII genes is tightly regulated, and epigenetics plays a crucial role. Indeed, different levels of histone modifications at the promoter region of MHCI and MHCII genes have been observed upon transcription activation, suggesting that histone modifiers might be associated with the MHC-enhanceosome (60–62). CIITA possesses *per se* a histone acetyltransferase (HAT) activity (63, 64) and has been shown to interact with chromatin remodeling factors such as histone lysine acetyltransferases p300/CREB-binding protein (CREBBP) and general control nonderepressible 5 (GCN5) (56, 57, 65). These two factors were also shown to enhance the transactivation activity of overexpressed NLRC5 (19, 49). Moreover, GCN5 has been recently reported to interact with and regulate NLRC5 nuclear retention (19). Yet, the mechanisms underlying the activity of NLRC5 and CIITA at the MHC-enhanceosome are most likely manifold and awaiting future investigation.

## NLRC5, CIITA, and butyrophilins

The best-studied activity of NLRC5 and CIITA is to control MHC genes’ expression and canonical antigen presentation pathways; however, additional targets have been identified that are regulated by CIITA or NLRC5 and a *bona fide* enhanceosome complex. Among these, *BTN* genes are of particular interest. Human *BTN* genes map to the telomeric end of the extended MHC locus, already suggesting a connection with MHC genes (66, 67). BTNs are transmembrane proteins exhibiting structural similarities to the B7 family of co-stimulatory molecules, which include the crucial immunomodulatory ligands PD-L1 and CD80 (67). The family member BTN2A2 indeed contributes to the control of T cell activation and proliferation and – accordingly – *Btn2a2-*knockout mice are more susceptible to T cell-mediated autoimmunity (68–72).

In 2016, Sarter et al. reported that CIITA transcriptionally regulates *BTN2A2* gene expression both in the human and the mouse system (69). An SXY consensus was identified at the *BTN2A2* promoter and the S-box sequence was compatible with CIITA-mediated regulation (8, 69).

The human BTN2A1, BTN3A1, BTN3A2, and BTN3A3 are emerging as key regulators of non-conventional Vγ9Vδ2 T cells, which are important effectors against selected microbial infections and cancers and have been shown to correlate with improved patient outcome when infiltrating tumoral tissue (73–85). Vγ9Vδ2 T cells sense and expand in response to so-called phosphoantigens (pAgs), metabolites of the isoprenoid synthesis pathway that accumulate in infected or transformed cells (**Figure 1**) (85). These metabolites, such as HMB-PP ((E)-4-hydroxy-3-methyl-but-2-enyl diphosphate), can be produced by parasitic and bacterial pathogens, including *Mycobacterium tuberculosis* or *Listeria monocytogenes* (86, 87). Isopentenyl pyrophosphate (IPP) is instead an endogenous form of pAg that accumulates in certain tumor cells (88). This is due to a dysfunctional mevalonate pathway, which is important for the biosynthesis of isoprenoid (terpenoid) molecules used in processes as diverse as cell membrane maintenance, protein modification, and synthesis of steroid hormones, coenzyme Q10, etc. (89). pAgs bind to the intracellular B30.2 domain, a multifunctional domain found in many innate immune sensors, of BTN3A1 (77, 90). This binding leads to a structural change in the complex formed by BTN3A1 and BTN2A1, which is required for the engagement of the Vγ9Vδ2 TCR, T cell proliferation, and cytotoxic attack of the abnormal cells (**Figure 1**) (83, 84, 91–95). In this context, BTN3A2 and BTN3A3 have been shown not only to increase the trafficking of BTN3A1 to the plasma membrane but also to be directly involved in the activation of Vγ9Vδ2 T cells (82, 94). Moreover, recent data hint at a crucial role for BTN3A3, which also possesses an intracellular B30.2 domain, in the activation of Vγ9Vδ2 T cells upon live *Listeria monocytogenes* infection (96). While the relevance of these BTN molecules has started to emerge over the last years, their exact role and dynamics await further elucidation (97).

Although BTNs and the associated Vγ9Vδ2 T cells are known to work in an MHC-independent manner, Dang et al. found a correlation between the MHC-regulating *NLRC5* and *BTN3A1–3* expression in cancerous cells and cells from healthy and *M. tuberculosis*-infected donors (8). Overexpressed NLRC5 was able to occupy and transactivate an atypical SXY module containing a reverse complement of the Y box, found in the promoter region of *BTN3A1–3* genes leading to enhanced Vγ9Vδ2 T cell-mediated elimination of tumoral cells (**Figures 1**, **2**). Recently, these data were confirmed and extended through a genome-wide CRISPR screen demonstrating the importance of endogenous NLRC5 for the expression of BTN3A1–3, and for cancer cells’ recognition and killing by Vγ9Vδ2 T cells (9, 98). This genome-wide screen also revealed a role for the histone acetylase CREBBP, an enzyme already found to promote the effects of NLRC5 and CIITA (49, 56). As CIITA is not found to play a major role in the activation of Vγ9Vδ2 T cells, these results support the idea that CREBBP is involved in NLRC5-dependent chromatin relaxation. This genetic screen also demonstrated the relevance of factors implicated in NLRC5/MHCI regulation, such as IRF1 and the enhanceosome factor RFXAP, for *BTN3A1–3* expression (9). This is in line with our research group data, obtained from cell lines bearing mutations in RFX factors (8), that indicate the involvement of an enhanceosome complex in the regulation of *BTN3A* gene transcription. Together, these findings underline the association between antigen and pAg presentation.

## Discussion – on the role of NLRC5 in the regulation of *BTN* and *BTN-like* genes

While the data discussed above clearly imply enhanceosome factors in the regulation of *BTN3A* gene transcription, the presence of a reverse complement Y box raises questions on the reciprocal orientation of the complex subunits. This entails a degree of flexibility that might be fulfilled either by changes in the architecture of the complex or by DNA looping, aspects that to date remain unexplored.

Moreover, the recently reported importance of IRF1 for *BTN3A1–3* gene expression and data indicating IRF1 binding proximal to the *BTN3A1–3* transcription start site led us to verify the presence of an ISRE sequence in their promoters (9). Indeed, an ISRE was found at the close distance and orientation relative to the SXY typical of the previously reported promoters regulated by NLRC5 (**Figure 2**) (44, 59). These notions suggest that, not only from a biological but also from a promoter docking point of view, combining IRF1 and NLRC5 might be important, strengthening the parallels between MHCI and *BTN3A* transcriptional control.

To better appreciate the regulation of Vγ9Vδ2 T cells’ activity, another key question concerns the mechanisms controlling *BTN2A1* transcription. Data from BLS-derived B cell lines indicate that *BTN2A1* expression is largely dependent on the RFX complex but not on CIITA (8). With regard to NLRC5, we did not witness its role in the transcriptional induction of endogenous *BTN2A1* and found just a weak correlation between *NLRC5* and *BTN2A1* transcript abundance in *M. tuberculosis* patients and ‘The Cancer Genome Atlas’ cancer samples (8). Interestingly, Liu and colleagues observed NLRC5-mediated transactivation of endogenous *BTN2A1* only upon EBV-reactivation, which also leads to IFNγ upregulation in nasopharyngeal carcinoma cells (98). These differences might imply a complex transcriptional regulation of *BTN2A1* at endogenous level, requiring the concomitant presence of NLRC5 together with additional transcriptional regulators, which – we might speculate – are stimulated by interferon. Indeed, an SXY module is present in the promoter of the *BTN2A1* gene and weak IRF1 binding upstream of the transcription start site has been recently reported (9), even if at the canonical ISRE position a strong consensus is not observed (**Figure 2**).

Questions arise also on the possible contribution by NLRC5 and CIITA in controlling the transcription of other members of the BTN/BTN-like (BTNL) family. Next to *BTN2A* and *BTN3A* genes, *BTN1A1*, *myelin oligodendrocyte glycoprotein (MOG)*, *erythroid membrane-associated protein* (*ERMAP)*, *BTNL2*, *3*, *8*, and *9* are found in the human genome (66, 99). Interestingly, *BTN1A1, MOG*, and *BTNL2* are located in the extended MHC locus on chromosome 6. Both BTN1A1 and BTNL2 have been reported to exert immunomodulatory functions and foster immune evasion when expressed on cancer cells (68, 100, 101) and *BTNL2* has been associated with immunological disorders such as sarcoidosis (102). Furthermore, recent work showed how BTNL3 as part of BTNL3/8 heterodimers binds to Vγ4 γδ TCR via germline-encoded regions, suggesting an interaction modality important to maintain homeostasis of this lymphocyte subset (91).

The high expression of BTN1A1 and BTNL3/8 molecules by non-immune cells – largely mammary and intestinal epithelial cells (66) – does not match the expected patterns of NLRC5- or CIITA-driven expression and recent results indicate that hepatocyte nuclear factor 4 and caudal type homeobox factors are crucial for the transcription of these *BTNL* genes, underlying the importance of tissue-specific transcription factors in their regulation (103). Moreover, available data did not reveal the presence of *BTN/BTNL* genes among the top targets regulated by NLRC5 or CIITA (8, 18, 34, 104, 105), with the exception of *MOG* – coding for a protein highly expressed in the brain – which has been identified as a putative CIITA and RFX5 target by chromatin immunoprecipitation (105). However, these results are limited to the analyzed tissue/cell type and promoter scanning for SXY motifs, which can be functional even when partly degenerated, remains challenging unless accompanied by experimental approaches. Therefore, these observations encourage further investigations on the transcriptional regulation of *BTN/BTNL* genes.

## Discussion – on the connections between NLRC5 and metabolism

MHC molecules are the core of vertebrates’ immune system, enabling the distinction between ‘self’ and ‘non-self’ and crucially promoting rejection or tolerance. NLRC5 regulates the transcription of MHCI, thus regulating cytotoxic T cell-mediated killing of infected and transformed cells. Intriguingly, recent results from others and us demonstrate that NLRC5 also controls the transcription of BTN3A1–3 molecules, which – together with BTN2A1 – are key for engaging Vγ9Vδ2 T cell-mediated killing of cells with a deregulated mevalonate pathway (**Figure 1**) (8, 9, 83, 84, 91–95). This reveals an unanticipated connection between the molecular circuits rendering cells presenting ‘non-self’ antigens and those with abnormal metabolic configurations detectable by the immune system.

Such a link is evolutionarily plausible as also an altered metabolism, albeit conforming to the definition of ‘self’, can represent a threat to the organism. Indeed, altered metabolism is central to malignant transformation. Along these lines, another target of NLRC5 is *H2-M3* (32, 34, 35), a murine non-classical MHC gene presenting N-formylated peptides that can either originate from prokaryotic pathogens or mitochondria (25, 27). The NLRC5-H2-M3 axis might thus support T cell immune responses against bacteria as well as toward cells with perturbed oxidative metabolism. Moreover, multiple studies described the connection between NLRC5 and metabolic conditions including obesity, but also an interaction of NLRC5 with PPARγ1, a key regulator of lipid metabolism and beyond (106), fostering the transcriptional activity of the latter (14, 107–110). Recent work also revealed that the acetyltransferase GCN5 interacts with NLRC5, promoting NLRC5 nuclear localization and activity (19). Notably, GCN5 acetylates PPARγ coactivator-1 (PGC-1), thereby suppressing its transcriptional activity, which is crucial for PPARγ-dependent transcription as well as for mitochondrial biogenesis (111). These notions support the hypothesis that the interaction of NLRC5 with GCN5 might represent a second mechanism modulating PPARγ activity, for example by indirectly favoring PGC-1 de-acetylation. Finally, an elegant study demonstrating a role for NLRC5 in inflammatory cell death upon heme sensing found that NLRC5 levels were primed by depletion of oxidized nicotinamide adenine dinucleotide (NAD^+^), a crucial cofactor in multiple cellular metabolic reactions (112). Based on these data, we can speculate that conditions characterized by reduced NAD^+^ levels, such as ageing or obesity (113), might increase NLRC5 levels to influence cellular metabolism or license cell death. How these conditions relate to the transcriptional role of NLRC5 remains an open and exciting question to explore. Altogether these observations add a layer of complexity to the already novel node represented by NLRC5 at the intersection between ‘non-self’, ‘deregulated-self’, and immunity and raise therefore NLRC5 to a multilevel modulator of metabolism, anti-microbial, and anti-cancer immune responses.

## Data availability statement

The original contributions presented in the study are included in the article, further inquiries can be directed to the corresponding author/s.

## Author contributions

GG: Writing – original draft, Writing – review & editing. FB: Writing – original draft, Writing – review & editing. SN: Writing – original draft, Writing – review & editing. JG: Writing – original draft, Writing – review & editing.
